# UV-Vis-NIR Broadband Dual-Mode Photodetector Based on Graphene/InP Van Der Waals Heterostructure

**DOI:** 10.3390/s25072115

**Published:** 2025-03-27

**Authors:** Mingyang Shen, Hao Liu, Qi Wang, Han Ye, Xueguang Yuan, Yangan Zhang, Bo Wei, Xue He, Kai Liu, Shiwei Cai, Yongqing Huang, Xiaomin Ren

**Affiliations:** 1 State Key Laboratory of Information Photonics and Optical Communications, Beijing University of Posts and Telecommunications, Beijing 100876, China; mingyangshen@bupt.edu.cn (M.S.); hliu@bupt.edu.cn (H.L.); han_ye@bupt.edu.cn (H.Y.); yuanxg@bupt.edu.cn (X.Y.); zhang@bupt.edu.cn (Y.Z.); weibo@bupt.edu.cn (B.W.); hexue@bupt.edu.cn (X.H.); kliu@bupt.edu.cn (K.L.); cswei@bupt.edu.cn (S.C.); yqhuang@bupt.edu.cn (Y.H.); xmren@bupt.edu.cn (X.R.); 2 School of Electronic Engineering, Beijing University of Posts and Telecommunications, Beijing 100876, China

**Keywords:** graphene/InP heterostructure, multifunctional photodetector, photovoltaic mode, photoconductive mode, broadband, self-powered, high-speed

## Abstract

Dual-mode photodetectors (DmPDs) have attracted considerable interest due to their ability to integrate multiple functionalities into a single device. However, 2D material/InP heterostructures, which exhibit built-in electric fields and rapid response characteristics, have not yet been utilized in DmPDs. In this work, we fabricate a high-performance DmPD based on a graphene/InP Van der Waals heterostructure in a facile way, achieving a broadband response from ultraviolet-visible to near-infrared wavelengths. The device incorporates two top electrodes contacting monolayer chemical vapor deposition (CVD) graphene and a bottom electrode on the backside of an InP substrate. By flexibly switching among these three electrodes, the as-fabricated DmPD can operate in a self-powered photovoltaic mode for energy-efficient high-speed imaging or in a biased photoconductive mode for detecting weak light signals, fully demonstrating its multifunctional detection capabilities. Specifically, in the self-powered photovoltaic mode, the DmPD leverages the vertically configured Schottky junction to achieve an on/off ratio of 8 × 10^3^, a responsivity of 49.2 mA/W, a detectivity of 4.09 × 10^11^ Jones, and an ultrafast response, with a rising time (τ_r_) and falling time (τ_f_) of 2.8/6.2 μs. In the photoconductive mode at a 1 V bias, the photogating effect enhances the responsivity to 162.5 A/W. This work advances the development of InP-based multifunctional optoelectronic devices.

## 1. Introduction

With the rapid advancements of modern information technology, broadband photodetectors (PDs) have found widespread applications in optical communications [1,2,3], sensor imaging [4,5,6], military reconnaissance [7,8], unmanned driving [9], and numerous other fields [10], increasingly becoming a focal point of research in both academia and industries. Traditional semiconductors, such as silicon (Si), gallium arsenide (GaAs), indium phosphide (InP), and their corresponding multicomponent alloys, exhibit suitable bandgap values as well as exceptional optical and electrical properties [11,12,13], making them highly suitable for the construction of broadband photodetectors. However, the majority of the reported photodetectors based on these semiconductors typically operate exclusively in either a photoconductive mode or photovoltaic mode [14,15,16,17,18,19,20], significantly limiting their potential for multifunctional detection capabilities.

Graphene, a prominent member of the 2D material family, is characterized by a monolayer state in which carbon atoms are arranged in a hexagonal lattice. It exhibits exceptional properties, including a zero bandgap, ultra-high carrier mobility, high conductivity, mechanical flexibility, and ease of functionalization [21,22], rendering it highly attractive for the development of advanced optoelectronic devices and garnering significant attention in the field. By combining graphene with traditional semiconductors, Van der Waals heterostructure photodetectors can be easily constructed, not only circumventing the intrinsic limitation of lattice matching required by conventional heterostructures but also significantly enhancing the detector’s photodetection performance and introducing novel photodetection functionalities. To date, a variety of photodetectors based on graphene/semiconductor heterostructures have been fabricated [23,24,25,26,27,28]. Benefiting from the semimetal (zero bandgap) nature of graphene, graphene/semiconductor heterostructures form Schottky heterojunction at their interfaces, creating a built-in electric field that facilitates the efficient separation of photogenerated carriers without the need for an external bias. As a result, photodetectors based on these heterostructures can operate in the photovoltaic mode, further enabling self-powered and high-speed photodetection.

Building on this foundation, researchers have discovered that, by designing appropriate electrodes for the graphene in graphene/semiconductor heterostructure photodetectors, photoconductive modes can be easily introduced alongside the photovoltaic mode, providing a practical solution for the development of dual-mode photodetectors (DmPDs). In the photoconductive mode, graphene serves as a fast transport channel for one type of photogenerated carrier, enabling high-responsivity photodetection through the photogating effect [29], which is particularly effective for detecting weak optical signals. Additionally, photodetectors working in the photoconductive mode can achieve bipolar light responses through control mechanisms such as regulating the gate voltage, wavelength, and light intensity, making them well-suited for applications in areas like optical communication, logic gates, and imaging [30]. The breakthrough of dual-mode photodetectors addresses the growing demand for multifunctional optoelectronic devices in next-generation photonic systems, where the simultaneous implementation of high-speed weak-signal detection (photoconductive mode) and self-powered operation (photovoltaic mode) is crucial for applications such as intelligent sensing networks [31]. For instance, DmPDs based on graphene/Si heterostructures [32,33] and graphene/InGaAs heterostructures [34] have been fabricated using a three-electrode design, integrating the advantages of both photodetection modes to achieve high-performance multifunctional photodetection. However, to the best of our knowledge, DmPDs based on graphene/InP heterostructures or even on other 2D material/InP heterostructures have not yet been reported, despite the fact that InP, with a direct bandgap energy of 1.34 eV and high carrier mobility, can absorb light with wavelengths ranging from ultraviolet-visible (UV-vis) to near-infrared (NIR) and enable high-speed photodetection.

In this study, a high-performance DmPD based on a graphene/InP Van der Waals heterostructure was successfully fabricated using a simple and efficient method. The device features two top electrodes that are exclusively in contact with monolayer chemical vapor deposition (CVD) graphene and a bottom electrode positioned on the backside of the InP substrate. The capability of flexible and rapid switching among these three electrodes enables the as-fabricated DmPD to work in either the self-powered photovoltaic mode or the biased photoconductive mode, while maintaining a broad spectral response ranging from UV-vis to NIR. In the self-powered photovoltaic mode, the graphene/InP heterostructure causes the DmPD to exhibit a competitive detection performance, including an on/off ratio of 8 × 10^3^, a responsivity of 49.2 mA/W, a detectivity of 4.09 × 10^11^ Jones, and an ultrafast response speed represented by a rising time (τ_r_) and falling time (τ_f_) of 2.8/6.2 μs. In the photoconductive mode at 1 V bias, the responsivity of the DmPD is significantly enhanced to 162.5 A/W (equivalent gain of 310), along with a detectivity of 1.76 × 10^10^ Jones and a τ_r_/τ_f_ of 61/4 ms. This work not only expands the diversity of InP-based heterostructure photodetectors but also provides a practical solution for fabricating multifunctional devices, contributing to the development of high-performance optoelectronic devices based on a Van der Waals heterostructure and being expected to play a significant role in the fields of multifunctional detection and intelligent optoelectronic sensing.

## 2. Materials and Methods

Materials: InP (100) substrate with thicknesses of 300 μm (*n*-type; dopant concentration: ~2 × 10^18^/cm^3^) was purchased from Beijing Tongmei Xtal Technology Co., Ltd (Beijing, China). Suspended monolayer chemical vapor deposition (CVD) graphene (*p*-type; size: 1 cm × 1 cm) was obtained from Hefei Vigon Material Technology Co., Ltd (Hefei, China).

**Figure 1:**
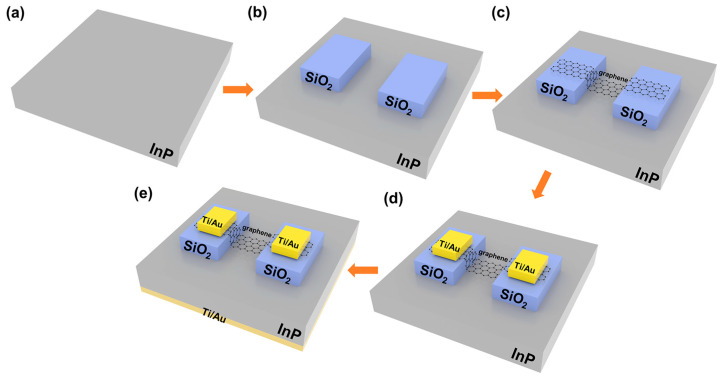
Flow chart illustrating the preparation process of the graphene/InP heterostructure DmPD. (**a**) The cleaning of the InP substrate. (**b**) The deposition of a 300 nm thick SiO_2_ insulating layer on the InP substrate. (**c**) The wet transfer of monolayer graphene to construct Van der Waals heterostructure with InP substrate. (**d**) The deposition of two top metal electrodes (Ti/Au, 50 nm/250 nm). (**e**) The deposition of a bottom metal electrode (Ti/Au, 50 nm/250 nm).

Fabrication of Graphene/InP heterostructure DmPD: First, the InP substrate was cut into 1 cm × 1 cm pieces that were cleaned in acetone, ethanol, and deionized water for 5 min each, and then dried with nitrogen (Figure 1a). Secondly, a 300 nm thick silicon dioxide (SiO_2_) dielectric layer was deposited on the InP substrate by plasma-enhanced chemical vapor deposition (PECVD), and then etched into two discrete rectangular patterns (300 μm × 300 μm) using a buffered oxide etchant (BOE) solution (HF: NH_4_F: H_2_O = 3 mL: 6 g: 10 mL), and the distance between the two patterns was 50 μm. (Figure 1b). Subsequently, monolayer CVD graphene was transferred onto the patterned InP substrate using a wet-transfer approach so as to form a graphene/InP Van der Waals heterostructure. The graphene was extended onto the two SiO_2_ patterns and then dry-etched into a rectangular shape (550 μm × 220 μm) using oxygen plasma (Figure 1c), while the area of the heterojunction zone was 50 μm × 220 μm. Next, two top electrodes (Ti/Au, 50 nm/250 nm) were deposited by physical vapor deposition (PVD), forming ohmic contact only with the graphene on the two SiO_2_ patterns (Figure 1d). Finally, a bottom electrode (Ti/Au, 50 nm/250 nm) was deposited on the backside of the InP substrate by PVD, completing the fabrication of the DmPD based on a graphene/InP Van der Waals heterostructure (Figure 1e). The as-fabricated DmPD operates in the photovoltaic mode when the bottom electrode and one top electrode are used, and switches to the photoconductive mode when both top electrodes (excluding the bottom electrode) are utilized.

Characterization and measurement: The morphology of the graphene/InP heterostructure DmPD was characterized using a field-emission scanning electron microscope (Zeiss Merlin, Germany). Raman analysis was performed using an inVia Raman spectroscope with a 532 nm laser source (Renishaw, UK). The absorption spectrum of the InP from 300 to 1200 nm was tested using the MStarter ABS system (Metatest, Nanjing, China). Optoelectronic characterizations were carried out using the ScanPro Advance system (Metatest, Nanjing, China), which includes a probe station, a broadband monochromator, and a 650 nm laser diode as the substitute light source. The fast light response was tested using a signal generator and oscilloscope. All measurements and characterizations were conducted at room temperature (300 K).

## 3. Results and Discussion

### 3.1. SEM Images and Raman Spectra Analysis

**Figure 2:**
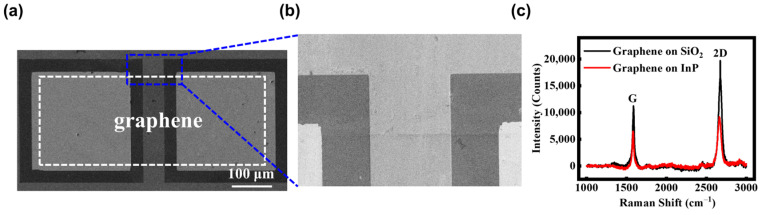
(**a**,**b**) A plane-view scanning electron microscope (SEM) image of a single graphene/InP heterostructure DmPD and its partially magnified view. (**c**) Raman spectra of graphene measured on a SiO_2_ pattern (black curve) and on the InP substrate (red curve), highlighting the characteristic peaks and shifts.

Figure 2a presents a plane-view scanning electron microscope (SEM) image of a single graphene/InP heterostructure DmPD. Figure 2b provides a magnified SEM image of the region between the two top electrodes shown in Figure 2a, and in it the top electrode, SiO_2_ pattern, InP substrate, graphene, and graphene/InP heterostructure can be easily distinguished. Additionally, Figure S1 presents a SEM image of a 4 × 3 array of as-fabricated DmPDs. Figure 2c illustrates the Raman spectra of graphene on a SiO_2_ pattern and the InP substrate, respectively. For the graphene on the SiO_2_ pattern, its 2D peak is observed at 2673.6 cm^−1^, and the G peak appears at 1586.1 cm^−1^. The intensity ratio of 2D/G is approximately 2, and the absence of defect-related peaks (e.g., D and D’) confirms that the graphene used is a high-quality monolayer with minimal defects [35]. For graphene on the InP substrate, the 2D and G peaks are located at 2662.4 cm^−1^ and 1582.9 cm^−1^, respectively, which is consistent with the values reported in the previous literature [36]. The slight Raman shifts are attributed to the variations in residual stress caused by the discrepancy in the template underneath the graphene.

### 3.2. Working Principle of Graphene/InP Heterostructure DmPD

**Figure 3:**
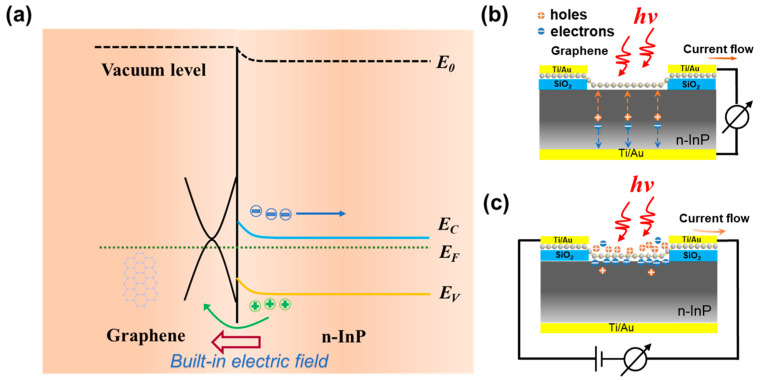
(**a**) Energy band diagram of the *p*-graphene/*n*-InP heterostructure without the need of external bias. (**b**,**c**) Schematics depicting the transportation of photogenerated carriers and the corresponding photocurrent direction when the graphene/InP heterostructure DmPDs operates in photovoltaic mode and photoconductive mode, respectively.

To elucidate the working principle of the graphene/InP heterostructure DmPD, we analyzed the energy band diagram of the heterostructure. The energy band diagram of the graphene/*n*-InP Schottky junction without external bias is plotted in Figure 3a. The work function value of the CVD-grown graphene (E_F_-_gr_) is shifted from its intrinsic value of 4.5 eV to approximately 4.8 eV below the vacuum level due to slight *p*-doping in the ambient air [37]. Since the InP substrate used is *n*-doped, its Fermi level (E_F_-_InP_) lies close to the conduction band (about 4.4 eV below the vacuum level [38]), indicating a work function value of approximately 4.4 eV for the InP. Upon forming the graphene/InP Schottky junction, some of the electrons transfer from the *n*-type InP into the graphene because of the Fermi level difference, which leads to upward bending of the electronic bands of InP at the graphene/InP interface and, hence, a built-in electric field directed from the InP towards the graphene is formed. When this Schottky junction is under illumination, excess holes and electrons are collected in the graphene and InP, respectively.

As depicted in Figure 3b, when the DmPD operates in the photovoltaic mode, a substantial number of photogenerated electron–hole pairs are generated once the graphene/InP Van der Waals heterostructure is illuminated by the incident light with an appropriate wavelength range. Most of these pairs are generated on the InP side of the heterostructure. Driven by the built-in electric field, these photogenerated electron–hole pairs are efficiently separated without the need for an external bias. The holes are swept to the graphene side, while the electrons migrate toward the *n*-InP substrate. These separated carriers are quickly collected by the top electrode and bottom electrode, respectively, generating a remarkable photocurrent.

When the DmPD is switched to the photoconductive mode, the built-in electric field generated by the graphene/*n*-InP Schottky junction remains and continues to separate the photogenerated electron–hole pairs. However, since the bottom electrode contacting the backside of the InP substrate is no longer utilized (as shown in Figure 3c), there is no current pathway to collect the separated electrons. As a result, the separated electrons will accumulate at the graphene/InP interface and will be unable to enter *n*-InP layer, leading to the trapping of holes in graphene at the graphene/InP interface, at which the majority of the carriers in the graphene are holes. In the absence of an applied bias across the two top electrodes connected solely to the graphene, these trapped holes rapidly recombine with the photogenerated electrons in the graphene. Due to the lack of an applied bias to drive carrier transport, the photocurrent in the graphene channel becomes negligible, rendering the DmPD incapable of self-powered operation in the photoconductive mode.

Once an external bias is applied across the two top electrodes, photogenerated holes in the graphene channel drift rapidly and are collected by the top electrode, subsequently flowing into the external circuit under the effect of the bias and thereby generating a significant photocurrent (Figure 3c). Simultaneously, holes from the bias source are immediately injected into the graphene channel to maintain the electrical neutrality. These injected holes drift back to the top electrode under the bias voltage, and this process repeats until the holes recombine with the previously trapped electrons at the graphene/InP interface. Owing to the ultrahigh carrier mobility inherent to graphene, each hole from a single photogenerated electron–hole pair is recirculated many times within the lifetime of the trapped electrons, effectively mimicking the simultaneous absorption of multiple photons, which results in the generation of multiple photogenerated electron-hole pairs and internal gain. This phenomenon, well-known as the photogating effect, imparts an exceptional photocurrent amplification as well as very high responsivity to the graphene/InP heterostructure DmPD when it is working in the biased photoconductive mode.

### 3.3. Optoelectronic Properties of the DmPD Working in Photovoltaic Mode

**Figure 4:**
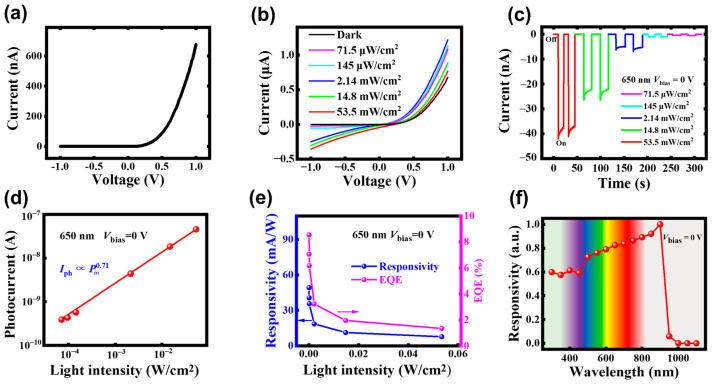
(**a**,**b**) Dark I–V curve and illuminated I–V curves of the graphene/InP heterostructure DmPD working in photovoltaic mode. (**c**) I–T curves of the DmPD in self-powered photovoltaic mode under 650 nm illumination with various intensities. (**d**,**e**) Dependence of photocurrent, *R*, and *EQE* on the incident light intensity in self-powered photovoltaic mode. (**f**) Spectral response of the DmPD in self-powered photovoltaic mode.

The dark current–voltage (I–V) curve of the DmPDs was measured and illustrated in Figure 4a. A pronounced asymmetry is clearly observed, with a rectification ratio exceeding 2 × 10^4^ within a voltage range of ±1 V, confirming the successful fabrication of a high-performance graphene/InP Van der Waals heterostructure. Subsequently, the optoelectronic properties of the as-fabricated graphene/InP heterostructure DmPDs were thoroughly assessed in the photovoltaic mode. To explore the influence of the incident light intensity on the DmPD, a series of photoresponse measurements were conducted. Figure 4b presents the I–V curves of the DmPD under 650 nm laser illumination (size of the light spot is 0.002 cm^2^), with light intensity ranging from darkness to 53.5 mW/cm^2^. A progressive increase in the current (*I*_light_) with a rising light intensity is very clear. Additionally, the *I*_light_ under reverse bias exhibits a distinct enhancement, highlighting the DmPD’s bias-dependent photoresponse. Figure 4c illustrates *I–T* curves of the DmPD in a self-powered state under the same 650 nm laser illumination, again with the light intensity varying from darkness to 53.5 mW/cm^2^. Here, the dark current (*I*_dark_) of 5 × 10^−12^ A is measured out (Figure S2a), and a maximum on/off ratio (*I*_light_/*I*_dark_) of 8 × 10^3^ is reached at 53.5 mW/cm^2^, demonstrating the DmPD’s robust photodetection capability. Further analysis of the relationship between the photocurrent *I*_ph_ (*I*_ph_ = *I*_light_ − *I*_dark_) and the incident light intensity is shown in Figure 4d. A power-law fitting of *I*_ph_ ∝ *P*_in_^γ^ yields a power exponent γ of 0.71, indicating the presence of a certain recombination losses within the investigated light intensity range.

The responsivity (*R*) and external quantum efficiency (*EQE*), two critical parameters for evaluating photodetector performance, can be calculated using the following formulas [39,40]:

(1) $$R=\frac{I_{\mathrm{ph}}}{P_{\mathrm{in}}}$$

(2) $$EQE=R\frac{h\cdot c}{\lambda \cdot q}$$

where *P*_in_ is the incident light power (the product of the incident light intensity and the device sensing area), *h* is the Planck constant, *c* is the speed of light in a vacuum, *λ* is the wavelength of the incident light, and *q* is the electron charge. As shown in Figure 4e, both the *R* and *EQE* exhibit inverse dependence on the illumination intensity in a self-powered state. This behavior arises because, as the incident light intensity increases, the number of photogenerated electron–hole pairs increases, leading to a higher carrier concentration. Consequently, the recombination rate of the photogenerated carriers also increases, resulting in a reduction in responsivity. Remarkably, the DmPD in the self-powered photovoltaic mode achieves a responsivity of 49.2 mA/W and an *EQE* of 9.39% under a minimum light intensity of 71.5 μW/cm^2^, demonstrating its excellent optoelectronic conversion efficiency even at zero bias.

The specific detectivity (D*) is another important parameter for photodetectors, and has an inverse relationship with the noise equivalent power (*NEP*). The *NEP* and D* are expressed as the following equation [41,42]:

(3) $$D^{*}=\frac{{\left(A\cdot B\right)}^{1/2}}{NEP}$$

(4) $$NEP=\frac{\sqrt{2qI_{\mathrm{dark}}}}{R}$$

where *A* is the device area and *B* is its bandwidth (typically set to 1 Hz and serves as a standard reference), while *R* is the responsivity under the minimum incident light intensity. While the *I*_dark_ is 5 pA (Figure S2a) and the *R* reaches a maximum value of 49.2 mA/W under a minimum incident light intensity of 71.5 μW/cm^2^, the *NEP* and D* are calculated to be 2.56 × 10^−14^ W·Hz^−1/2^ and 4.09 × 10^11^ Jones, respectively.

The spectral response of the DmPD in the self-powered photovoltaic mode was characterized with a monochromator to provide incident light with a constant intensity across the range of 300–1100 nm in 50 nm increments. As shown in Figure 4f, the DmPD exhibits a pronounced responsivity across the 300–900 nm spectral range, followed by a sharp decline for wavelengths exceeding 950 nm. The observed cutoff wavelength shows good agreement with the bandgap energy of InP. We also tested the absorption spectrum of InP, as shown in Figure S3, which directly confirms InP as the primary light-absorbing medium governing the photovoltaic response. Given the extremely low absorption coefficient of the monolayer graphene across the entire spectrum, its contribution to the photocurrent is negligible in this mode.

**Figure 5:**
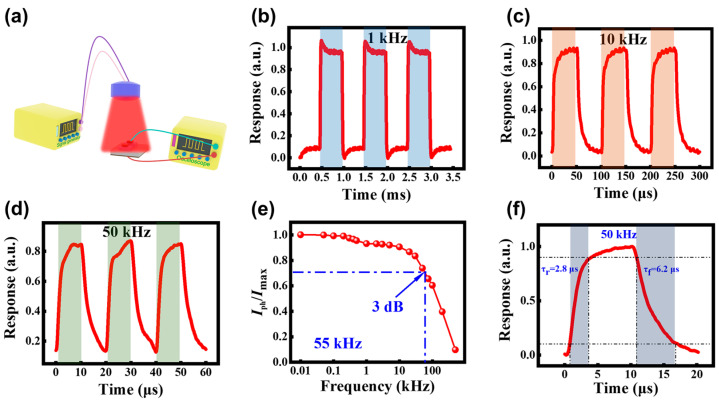
(**a**) Schematic diagram of the experimental setup for characterizing the photoresponse. Photoresponse of the graphene/InP heterostructure DmPD in self-powered photovoltaic mode to 650 nm pulsed light irradiation at switching frequencies of (**b**) 1 kHz, (**c**) 10 kHz, and (**d**) 50 kHz. (**e**) Relative balance (*I*_ph_/*I*_max_) versus switching frequency, indicating a 3 dB cutoff frequency of ~55 kHz achieved by the DmPD in self-powered photovoltaic mode. (**f**) A single cycle of normalized light on/off response at 50 kHz, used to determine the rising time (τ_r_) and falling time (τ_f_) of the DmPD in self-powered photovoltaic mode.

Owing to the graphene/InP Van der Waals heterostructure, the as-fabricated DmPDs wereexpected to exhibit the ultra-fast response capability in photovoltaic mode. This capability was demonstrated under 650 nm illumination with certain switching frequencies, with the experimental measurement system illustrated schematically in Figure 5a. Figure 5b−d present the zero-bias transient photoresponse at switching frequencies of 1 kHz, 10 kHz, and 50 kHz, respectively. By analyzing the normalized photocurrent decay (*I*_ph_/*I*_max_) curve as a function of the switching frequency, a 3 dB bandwidth of approximately 55 kHz can be deduced, as shown in Figure 5e. The response speed is further evaluated by examining the rising and falling edges of a single light on/off cycle. The time intervals during which the response rises from 10% to 90% and falls from 90% to 10% of its peak current were defined as the rising time (τ_r_) and the falling time (τ_f_), respectively [43]. As shown in Figure 5f, the DmPD achieves a τ_r_ of 2.8 μs and a τ_f_ of 6.2 μs at zero bias, fully showcasing its strong competitiveness among photodetectors based on 2D material/InP heterostructures.

**Figure 6:**
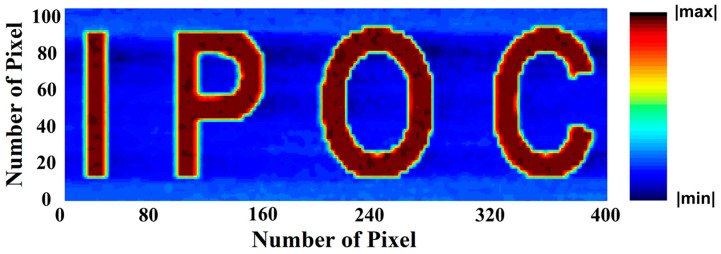
Image sensing demonstration using a single graphene/InP heterostructure DmPD working in self-powered photovoltaic mode. A clear current mapping of the “IPOC” image under 650 nm illumination is successfully captured.

To evaluate the imaging capabilities of the graphene/InP heterostructure DmPD in the self-powered photovoltaic mode, a single-pixel detector imaging system was built. A 650 nm wavelength laser beam was directed onto an image mask patterned with the “IPOC” logo (IPOC stands for State Key Laboratory of Information Photonics and Optical Communications) and the transmitted beam illuminated the DmPD. By moving the mask, the resulting spatially resolved photoreponse was recorded using a semiconductor analyzer connected to the DmPD. After real-time data acquisition and processing by a computer, the “IPOC” logo was clearly reconstructed (as shown in Figure 6), thereby demonstrating the excellent imaging capability of the DmPD in the photovoltaic mode. This capability is attributed to the DmPD’s ultralow dark current (on the order of 10^−12^ A) and high photocurrent generation, underscoring the potential of the device for applications in high-speed, low-power-consumption optical imaging systems.

### 3.4. Optoelectronic Properties of the DmPD Working in Photoconductive Mode

**Figure 7:**
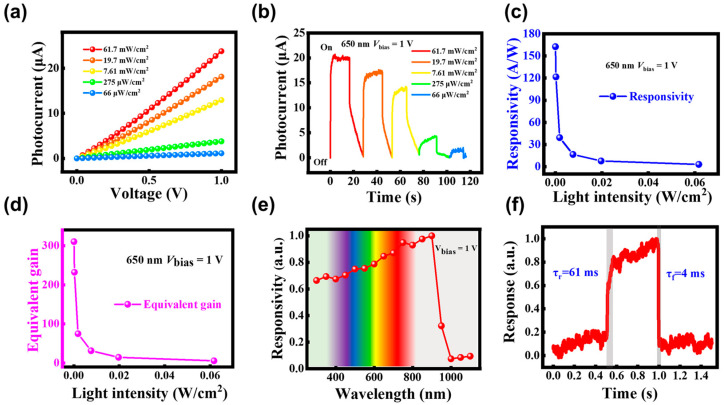
(**a**,**b**) *I*_ph_–*V* curves (**a**) and *I*_ph_–*T* curves (**b**) of the DmPD working in photoconductive mode under 650 nm light illumination at varying intensities, measured at 1 V bias. (**c**,**d**) The calculated responsivity (**c**) and equivalent gain (**d**) as a function of light intensity. (**e**,**f**) Spectral response (**e**) and response speed analysis (**f**) of the graphene/InP heterostructure DmPD working in photoconductive mode at 1 V bias.

When the two top electrodes in contact with the graphene are selected to connect with the probes, the DmPD switches to operate in the photoconductive mode. Figure 7a shows the photocurrent–bias voltage (*I*_ph_–*V*) curves of the DmPD under 650 nm illumination with various light intensities in the photoconductive mode, revealing an approximately linear relationship between the bias voltage and the photocurrent. Figure 7b presents the photocurrent–time (*I*_ph_–*T*) curves at 1 V bias under the same illumination conditions. Owing to the photogating effect enabled by the graphene/InP heterostructure, the device can generate a photocurrent on the order of 1 μA even when the incident light intensity is reduced to the μW/cm^2^ level. In contrast, the DmPD working in the self-powered photovoltaic mode generates a photocurrent on the order of only 1 nA when exposed to the same light intensity level. Clearly, there is a nearly three-orders-of-magnitude enhancement in the responsivity of this mode compared to the photovoltaic mode, demonstrating the suitability of the DmPD in the photoconductive mode for weak-light detection scenarios. The *R* and *EQE* of the DmPD in the photoconductive mode were calculated using Equations (1) and (2), as shown in Figure 7c,d. Both the *R* and *EQE* increase as the incident light intensity decreases, reaching a maximum *R* of 162.5 A/W and an *EQE* of 310 (which can be equivalently interpreted as the gain) under a minimum light intensity of 66 μW/cm^2^, which are far higher values than those achieved in the self-powered photovoltaic mode. The (*NEP*) and D* were derived from the dark current at 1 V bias (Figure S2b) using Equations (3) and (4). The calculated D* of 1.76 × 10^10^ Jones in the photoconductive mode is lower than that achieved in the photovoltaic mode, primarily due to the higher noise level at the 1 V bias in this mode.

Figure 7e gives the spectral response of the DmPD working in the photoconductive mode at 1 V bias, which exhibits a broadband detection range spanning from 300 nm (UV) to 1100 nm (NIR), which is broader that that observed in the self-powered photovoltaic mode. Notably, the spectral response persists at wavelengths exceeding 950 nm, primarily due to the photocurrent generated by the graphene alone as an absorbing layer in the long-wavelength region of the DmPD in the biased photoconductivity mode, which is consistent with the energy band analysis presented in Figure 2. In Figure 7f, the response speed of the DmPD in the photoconductive mode at 1 V bias is also characterized by the rising time (τ_r_) and the falling time (τ_f_), respectively. As shown in Figure 7f, the measured τ_r_/τ_f_ is 61/4 ms, which is approximately three orders of magnitude slower compared to that in the self-powered photovoltaic mode.

### 3.5. Performance Comparison of Graphene/InP Heterostructure Photodetectors

To comprehensively assess the performance of the fabricated graphene/InP heterostructure DmPD, we compiled and analyzed key parameters from the recent literature on various graphene/InP heterostructure photodetectors [43,44,45,46,47,48], comparing them with the as-fabricated DmPD, as outlined in Table 1. Notably, the existing graphene/InP heterostrucutre photodetectors are limited to operating solely in the photovoltaic mode, while our DmPD demonstrates an overwhelming advantage with its dual-mode photodetection capability. Particularly, in the self-powered photovoltaic mode, our DmPD exhibits a faster response speed than most other graphene/InP heterostructure photodetectors. Moreover, in the biased photoconductive mode, the responsivity of our DmPD ranks among the highest reported values.

Overall, our DmPD shows the fastest response speed and the highest D* compared to other reported graphene/Si and graphene/InGaAs heterostructure DmPDs in the photovoltaic mode, which is mainly due to the high carrier mobilities of graphene and InP. However, the responsivity of ours is lower than that of other existing DmPDs. In the photoconductive mode, the response speed and D* are comparable to those of previously reported graphene-based DmPDs, but the responsivity is lower. The lower responsivity in both the photovoltaic and photoconductive modes is due to carrier recombination within the heterostructure, which results in a loss of photogenerated carriers and reduced overall responsivity.

## 4. Conclusions

In summary, we have successfully fabricated a DmPD based on a graphene/InP Van der Waals heterostructure and three electrodes using a simple fabrication process, achieving a broad spectral response ranging from UV-vis to infrared. By switching the working electrodes, the DmPD can operate in either the self-powered photovoltaic mode or biased photoconductive mode, demonstrating competitive detection performance compared to other InP-based photodetectors. In the photovoltaic mode, benefiting from its graphene/InP heterostructure, the DmPD exhibits a noticeable rectification ratio of 2 × 10^4^, a dark current as low as 5 pA, a high on-off ratio of 8 × 10^3^, a maximum responsivity of 49.2 mA/W, an *EQE* of 9.39%, a detectivity of 4.09 × 10^11^ Jones, and an ultra-fast response speed represented by a rising time (τ_r_) and falling time (τ_f_) of 2.8/6.2 μs, all achieved in a self-powered state, fully demonstrating the advantage of not relying on an external power source and high-speed photodetection. In the photoconductive mode, under only 1V reverse bias, the device achieves a much higher responsivity of 162.5 A/W (equivalent to a gain of 232), a detectivity of 1.76 × 10^10^ Jones, and a τ_r_/τ_f_ of 61/4 ms, demonstrating its potential for detecting ultra-weak light. The as-fabricated DmPD is well-suited for a variety of detection and imaging applications, and even enables advanced functionalities. Furthermore, this work provides a solution for fabricating InP-based multifunctional photodetectors and makes certain contributions to the development of high-performance and intelligent optoelectronic devices.

## Figures and Tables

**Table 1:** Comparison of graphene/InP heterostructure photodetectors in terms of key photodetection performance.

Device Structure	Measurement Conditions	R (A/W)	D* (Jones)	τ_r_/τ_f_	Ref.
Graphene/*n*-InP heterostructure DmPD (Photovoltaic mode)	V_bias_ = 0 V	0.049	4.09 × 10^11^	2.8 μs/6.2 μs	This work
	λ = 650 nm				
Graphene/*n*-InP heterostructure DmPD (Photoconductive mode)	V_bias_ = 1 V	162.5	1.76 × 10^10^	61 ms/4 ms	This work
	λ = 650 nm				
Graphene/*n*-InP photodetector	V_bias_ = 0 V	0.046	3.62 × 10^9^	25.9 μs/43.3 μs	[43]
	λ = 980 nm				
Graphene/*n*-InP with silica @AuNR photodetector	V_bias_ = 0 V	0.140	1.1 × 10^10^	441 ns/433 ns	[44]
	λ = 980 nm				
Graphene/*p*-InP/Al_2_O_3_ photodetector	V_bias_ = 0 V	0.002	1.2 × 10^10^	/	[45]
	λ = 808 nm				
SWCNT/graphene/Al_2_O_3_/*p*-InP photodetector	V_bias_ = 0 V	0.154	1.3 × 10^12^	40 μs/180 μs	[46]
	λ = 808 nm				
Ag NPs/ graphene/*p*-InP photodetector	V_bias_ = 0 V	0.008	1.2 × 10^10^	246.8 μs/800 μs	[47]
	λ = 808 nm				
PbS (TBAI)/Graphene/ Al_2_O_3_/*p*-InP photodetector	V_bias_ = 1.1 V	0.145	9.7 × 10^10^	11 μs/76 μs	[48]
	λ = 808 nm				

## Data Availability

The data that support the findings of this study are available from the corresponding author upon reasonable request.

## Supplementary Materials

The following supporting information can be downloaded at: https://www.mdpi.com/article/10.3390/s25072115/s1, Figure S1: SEM image of 4 × 3 array of the graphene/InP van der Waals heterostructure DmPDs; Figure S2: Dark I-T curves of graphene/InP van der Waals heterostructure DmPDs working in the photovoltaic mode at zero bias and (**b**) in photoconductive mode at 1 V bias. Figure S3: Absorption spectrum of InP ranging from 300 to 1200 nm.
