# Supplementary material for: UV-Vis-NIR Broadband Dual-Mode Photodetector Based on Graphene/InP Van Der Waals Heterostructure
Source: Sensors (Basel). 2025 Mar 27;25(7):2115. doi: 10.3390/s25072115 (PMC11991182; doi:10.3390/s25072115)
Supplement: Supplementary file 1 [file sensors-25-02115-s001.zip › sensors-3498639-supplementary.pdf]

Supporting Information:

UV-Vis-NIR Broadband Dual-Mode Photodetector Based on Graphene/InP Van der Waals Heterostructure

Mingyang Shen [a,b], Qi Wang [a,b,*], Hao Liu [a,b], Han Ye [a,b], Xueguang Yuan [a,b], Yangan Zhang [a,b], Bo Wei [a,b], Xue He [a,b], Kai Liu [a,b], Shiwei Cai [a,b], Yongqing Huang [a,b] and Xiaomin Ren [a,b]

a State Key Laboratory of Information Photonics and Optical Communications, Beijing University of Posts and Telecommunications, Beijing 100876, People's Republic of China
b School of Electronic Engineering, Beijing University of Posts and Telecommunications, Beijing 100876, People's Republic of China
* Correspondence: Corresponding author: wangqi@bupt.edu.cn

Supplementary Figures:

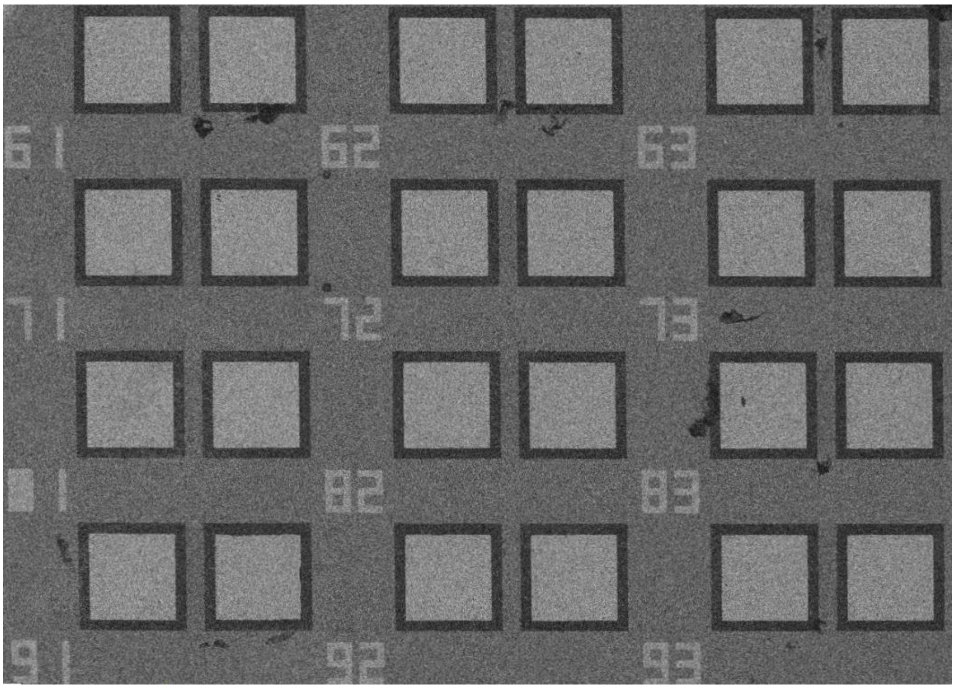

Figure S1. SEM image of 4×3 array of the graphene/InP van der Waals heterostructure DmPDs.

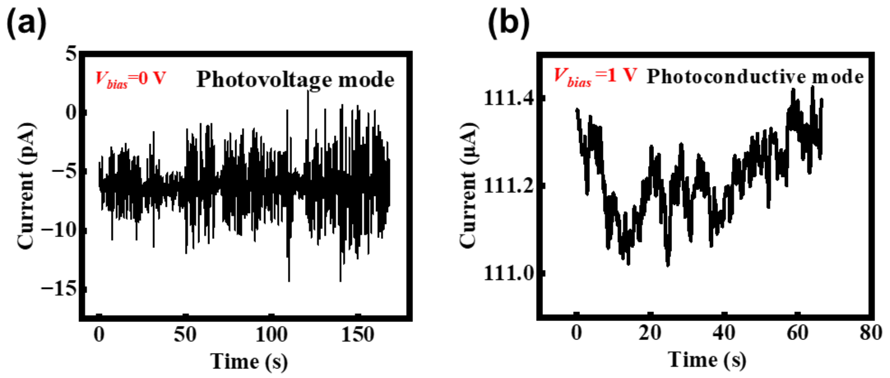

Figure S2. (a) Dark *I-T* curves of graphene/InP van der Waals heterostructure DmPDs working in the photovoltaic mode at zero bias and (b) in photoconductive mode at 1 V bias.

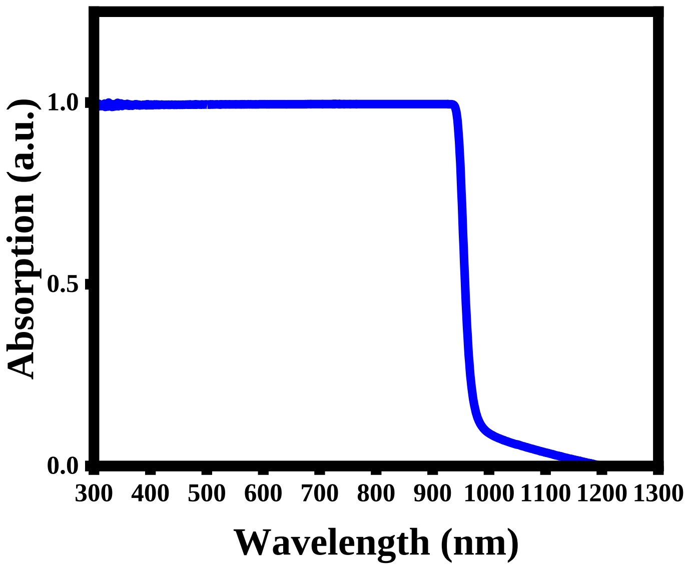

Figure S3. Absorption spectrum of InP ranging from 300 to 1200 nm.
